# Cadaveric Bone Donation amid COVID-19 Pandemic: An Unstoppable Chronicle in Uncharted Waters

**DOI:** 10.5704/MOJ.2111.028

**Published:** 2021-11

**Authors:** A Mansor, S Ramalingam, N Yusof

**Affiliations:** Department of Orthopaedic Surgery (NOCERAL), University of Malaya, Kuala Lumpur, Malaysia

Dear editors,

We would like to share a scenario in cadaveric bone donation for bone banking activities after the emergence of Coronavirus 2 (COVID-19) in early March 2020. COVID-19 is the genesis for an ongoing pandemic of respiratory illness, which is affecting potential donation in the country. As warned by World Health Organization (WHO), possible modes of transmission are close contact, droplet, airborne, fomite, faecal-oral, blood-borne, mother-to-child and animal-to-human. To date, there is no report on donor-related COVID-19 infections^1^. The risk of developing COVID-19 from Severe Acute Respiratory Syndrome Coronavirus (SARS-CoV-2) infected cadaveric donor is still unknown. Therefore, tissue donation must adhere to strict protocols and extreme precaution is necessary when considering cadaveric donors for bone donation.

Our bone bank supplies radiation sterilised long bone allografts for various orthopaedic surgeries namely arthroplasty, oncology and trauma. These long bones are obtained from cadaveric donors identified and screened by the National Coordinators of the National Transplant Resource Centre (NTRC), Ministry of Health.

## Pre COVID-19 era (before March 2020)

In normal practice, cadaveric donors are identified after they are confirmed either as brain or cardiac dead, classified as donation after brain death (DBD) or donation after cardiac death (DCD), respectively. The NTRC screens the medical records and social history of the identified donor after consent is obtained from the next-of-kin.

**Fig. 1:**
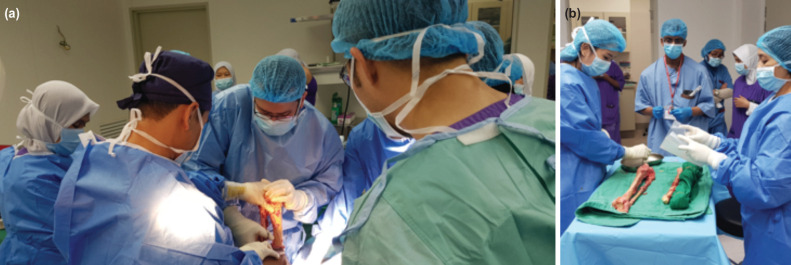
(a) Bone procurement is conducted in an operating theatre. (b) Packing of each procured bone into triple layer of plastic-linen-plastic.

Prior to procurement, Tissue and Organ Procurement (TOP) team of a particular hospital where the donor has been identified further screens the body to identify the donor’s suitability as a bone donor. Bone procurement is conducted in an operating theatre by the TOP team (Fig. 1a). Strict screening procedures entail stringent laboratory investigations to rule out the transmission of diseases to recipients. Bone swabs for a bacteriological test must have negative bacterial growth showing absence of microorganisms neither from the bone itself nor from the environment during procurement, while the donor’s blood for serological tests must be free from Hepatitis B, Hepatitis C, HIV and Syphilis. Each procured bone is individually packed in three layers of plastic-linen-plastic to avoid cross contamination (Fig. 1b). The donated bones are immediately sent to the bank, quarantined at temperature below -40° Celsius while the Donor Screening Report and laboratory test results received from the NTRC are reviewed in line with international standards. Only bones with negative results are accepted and sent for a sterilisation process using gamma radiation. Sterile bone allografts with graft identifications are stored in a deep freezer at -80° Celsius before suppling to hospitals.

## Current COVID-19 era (after March 2020 until now)

Under the new norm, COVID-2 test is included on top of the stringent donor screening with bacteriological and serological tests, to ensure the donated bone is free from the life threatening virus. All potential donors are tested for SARS-Cov-2 by taking nasopharyngeal and oropharyngeal swabs that are analysed by real-time reverse transcription polymerase chain reaction (rRT-PCR) test. Bone donation should be suspended from those who are tested positive. According to recent guidelines from National Health Service Blood Transplant (NHSBT) United Kingdom, donors who are at low-risk or tested negative are considered likely safe for donation^2^. As the recent variant is airborne and fast spreading, the TOP team is cognisant of potential spread of COVID-19 during procurement by taking all the safety measures to prevent it at all costs.

The bank received 18 long bones from 4 cadaveric donors in 2019 (pre-Covid era) and 22 bones from 5 donors in 2020, of which 3 were procured after March 2020 (during the pandemic). The bone donation programme has been suspended temporarily in 2021 due to outbreaks where COVID-19 cases which need utmost attention were increasing.

Bone donation is truly an act of altruism and empathy from the donors who have a deep desire to donate and help others in need. Adaptability to emerging knowledge is crucial when uncertainties occur in the field of donation and to have a continuous life-saving practice in this country. The trust given to bone bankers to practice high standards of bone banking amidst the pandemic must be well observed thus ensuring the safety of the allografts as well as the bank personnel.
